# Chemisorption of iodine-125 to gold nanoparticles allows for real-time quantitation and potential use in nanomedicine

**DOI:** 10.1007/s11051-017-3840-8

**Published:** 2017-04-19

**Authors:** Adrian A Walsh

**Affiliations:** 1 Translational Medicine, Liverpool University, Ashton Street, Liverpool, L69 3GE UK; 2 Nano Biosols Ltd, Liverpool Science Park, 131 Mount Pleasant, Liverpool, L3 5TF UK

**Keywords:** Gold nanoparticles, Iodine-125, Radioactive labelling, Quantitation, Chemisorption, Nanomedicine

## Abstract

Gold nanoparticles have been available for many years as a research tool in the life sciences due to their electron density and optical properties. New applications are continually being developed, particularly in nanomedicine. One drawback is the need for an easy, real-time quantitation method for gold nanoparticles so that the effects observed in in vitro cell toxicity assays and cell uptake studies can be interpreted quantitatively in terms of nanoparticle loading. One potential method of quantifying gold nanoparticles in real time is by chemisorption of iodine-125, a gamma emitter, to the nanoparticles. This paper revisits the labelling of gold nanoparticles with iodine-125, first described 30 years ago and never fully exploited since. We explore the chemical properties and usefulness in quantifying bio-functionalised gold nanoparticle binding in a quick and simple manner. The gold particles were labelled specifically and quantitatively simply by mixing the two items. The nature of the labelling is chemisorption and is robust, remaining bound over several weeks in a variety of cell culture media. Chemisorption was confirmed as potassium iodide can remove the label whereas sodium chloride and many other buffers had no effect. Particles precoated in polymers or proteins can be labelled just as efficiently allowing for post-labelling experiments in situ rather than using radioactive gold atoms in the production process. We also demonstrate that interparticle exchange of I-125 between different size particles does not appear to take place confirming the affinity of the binding.

## Introduction

Gold nanoparticles have been in use as a research tool in the life sciences for over 40 years. The original application was that of an electron dense marker for immunoelectron microscopy (Faulk and Taylor 1971). Antibodies coupled to colloidal gold bind to the antigenic site on the biological specimens and disclose the location of the binding site by a black punctate dot visible in the electron microscope (Horisberger and Rossett 1977; Roth et al. 1978; Batten and Hopkins 1979; Slot and Geuze 1981).

The second wave of applications of gold nanoparticles developed when the optical properties of these nanomaterials were exploited in a number of in vitro assays whereby the macroscopic signal generated by the red colour of gold nanoparticles was utilised. Early examples were in light microscopy (Dewaele et al. 1983), protein blot staining (Moeremans et al. 1985), sol particle immunoassays (Leuvering et al. 1983) and in lateral flow diagnostic tests (Valecha et al. 1998; Sang et al. 1998). Other assays have exploited surface plasmon resonance properties by using the colour shift from red to blue when the nanoparticles are aggregated (Aslan et al. 2004). Surface-enhanced Raman scattering (SERS) (Grubisha et al. 2003; Shultz 2003) is another technique that exploits the spectroscopic properties of nanoparticles. DNA assays have also been developed utilising the optical properties of gold nanoparticles (Stofhoff et al. 2004) which can also be silver enhanced for greater sensitivity (Nam et al. 2004).

The third wave of applications of gold nanoparticles is currently underway in the form of nanomedicine, i.e. the use of nanomaterials for medical applications of imaging, diagnosis and therapy. Several publications have shown how the nanomaterials can be exploited to generate potential therapeutic actions. Hirsch et al. (2003) demonstrated how irradiating cells with infrared light that had been treated with gold nanoparticles made them more susceptible to hyperthermic destruction than with IR light alone. Hainfeld et al. (2004) showed how gold nanoparticles can enhance the destructive effect of conventional radiotherapy by interacting with the X-rays to produce more destructive secondary radiation. Other forms of therapy using gold nanoparticles include drug delivery (Cheng et al. 2008; Patra et al. 2009; Jain 2011). The imaging properties of gold have been suggested, exploiting its density as being potentially useful as a contrast agent (Zhang et al. 2009; Hainfeld et al. 2006). There are several recent review articles detailing the theranostic dual action of gold nanoparticles as both diagnostic and therapeutic in nanomedicine (Jain et al. 2012; Dreaden et al. 2012; Khlebtsov et al. 2013; Chen et al. 2016).

One drawback with the use of gold nanoparticles in nanomedicine is the lack of real-time quantitation of particle uptake and thus a failure to relate any beneficial or toxic effect to a known amount of material. The therapeutic efficiency of these nanomaterials is hard to calculate. The most sensitive method of quantifying gold nanoparticle uptake is inductively coupled plasma mass spectrometry (ICPMS), but this requires taking samples to be evaluated later (Myllynen et al. 2008; Scheffer et al. 2008; Allabashi et al. 2009). It is very sensitive and can be used retrospectively to analyse particle uptake but is not suitable for clinical work.

Use of radioactive gold, Au-198, has been tried (Hong et al. 2009; Chanda et al. 2010; Kannan et al. 2012), but this requires handling radioactive materials from the outset in the preparation of the nanoparticles and subsequent bio-functionalisation.

Another novel method for quantifying gold nanoparticles is based on fluorescence quenching assays (Aggarwal and Dobrovolskaia 2010). The metal particles sample must first be treated to break down the gold colloid into gold (111) which is then reacted with a fluorescent dye resulting in quenching by the gold. The amount of quenching is proportional to the gold concentration. Cell uptake experiments have been monitored by this method and can be conducted in a 96-well format.

Conventional cell labelling experiments using light microscopy in life science research is often carried out by immunofluorescence but gold nanoparticles labelled with a fluorescent tag cannot be tracked by this method due to quenching of fluorescence by the metal particles.

Development of a post-labelling technique with a suitable radionuclide would be more advantageous. What is required is a simple method that allows for real-time quantification or imaging of the nanoparticles in a preclinical setting which allows the efficiency of cell uptake studies or the localisation of particles in animal models to be fully assessed. If these nanomaterials are to be used in future targeted therapies, they should be easily imaged to assist the clinicians in their chosen therapy.

Thirty years ago, as part of a research team developing the use of colloidal gold particles as an imaging agent in immunoelectron microscopy, we developed a way of quantifying gold particles by radiolabelling the gold with iodine-125 (Beardmore et al. 1987). The paper demonstrated that uptake of I-125 gold particles could be tracked and quantified during cell uptake experiments. The potential of the technique was not fully exploited at the time as the use of radiolabelled probes was not desirable in immunoelectron microscopy due to the hazardous nature of the material. However, with the current development of potential nanomedical applications of gold nanoparticles a further analysis of the nature and properties of the association of iodine with gold nanoparticles is overdue. This publication updates our understanding, first described 30 years ago, of the nature of chemisorption of I-125 to gold nanoparticles in detail to help exploit this technique as a quantitative tool in nanogold research.

## Experimental section

### Materials

Chloroauric acid (HAuCl_4_·3H_2_O) was obtained from Aldrich. Iodine-125 (1 mCi; specific activity 17 Ci/mg; concentration 100 mCi/ml) was purchased from Perkins-Elmer. Magnetic beads (Protein A Mag Sepharose) were purchased from GE Healthcare. Sepharose beads and all proteins used for gold stabilisations and all other chemicals were obtained from Sigma.

### Preparing gold nanoparticles and conjugates

Fifteen-nanometre gold nanoparticles were made using the citrate method (Frens 1973). Five-nanometre particles were made by the method (Slot and Geuze 1985). For simplicity, both preparations are referred to as 5 and 15 nm in this publication although mean sizes are shown in the “Results” section. Nanoparticles were sized by analysing images obtained by TEM. Absorption spectra were produced between 400 and 600 nm using a UV-Vis spectrophotometer. Stabilisation of gold particles was done by the adsorption isotherm method where optimal pH and protein concentration were established (Geoghegan 1988). Typically 10 μg of protein were required to stabilise 1 ml gold nanoparticles. Unbound protein was removed by centrifugation washes. Nanoparticles were sized by TEM.

### Loading iodine-125 (I-125) on gold nanoparticles and desorption studies

Typically 1 μl iodine-125 (100 μCi) was diluted in 2 ml deionised water and used as a working stock solution. A standard curve for quantification of I-125 was made by taking increasing volumes of stock solution and counting the activity in a gamma counter. A linear standard curve could be drawn up to 50,000 cps. Loading gold with I-125 was achieved by simply adding the required amount of I-125 from the diluted stock solution to the prepared gold nanoparticles (1 ml, OD_520_ = 1) to allow chemisorption to take place. The final concentration of iodine in these preparations was approximately in the range 0.5 to 15 nM depending on the experiment. Binding was allowed for 30 min before centrifugal washing (15,000 rpm × 20 min for 15 nm gold). To study the effect of iodine adsorption on the nanoparticle spectral properties, cold iodide was added at tenfold increased concentrations from 2 nM to 20 μM, a total of four orders of magnitude. The absorption spectra were recorded for each increase in iodide concentration. Desorption experiments were undertaken with either β-mercaptoethanol or potassium iodide solutions at various concentrations as described in the “Results” section.

### Theoretical calculation of numbers of gold nanoparticles per preparation

The theoretical calculation of the number of gold nanoparticles in each preparation was based on gravimetric measurements using the method of Shang and Gao (2014) in their review of methods for nanoparticle quantitation. In order to calculate the number of gold nanoparticles per preparation certain assumptions have to be made. Assuming that there is a known mass of gold used in the preparation and that the density of gold is 19.3 g/cm^3^, then the total volume of particles combined can be calculated as *d* = *m*/*v*. Assuming that all the gold reactants are used and incorporated into roughly spherical particles of known diameter, then it is possible to calculate how many particles are present in total and thus their concentration as particles per millilitre. It is possible to test for completion of the reaction of gold ions into nanoparticles by pelleting the nanoparticles by centrifugation and adding a few grains of sodium borohydried to the clear supernatant. A pinkish to red colour indicates the presence of unused gold ions whereas a clear supernatant indicates completion of the reaction. Another point to consider is that the mass of gold in the final product is not the mass of gold chloride used in the reaction. In these preparations, the gold content was 51% of the gold chloride by mass as indicated by the supplier.

### Theoretical calculation of iodine atoms bound per gold particle

For 1 μCi of I-125 (17 Ci/mg), it was calculated that approximately 50 pg iodide ions were present. Given the calculated number of gold particles/ml and using the Avogadro constant (6.022 × 10^23^) for iodide estimation, it was possible to estimate the bound ratio of iodine to gold particles.

### Stability tests

A preparation of gold nanoparticles stabilised with Protein A and labelled with I-125 was stored in three different buffers for over 100 days at 5 °C. At regular time intervals, aliquots were taken and centrifuge washed to calculate the fraction of iodine still bound to the gold.

### Interparticle ligand exchange

Fifteen-nanometre gold particles (0.5 ml, not coated) but labelled with 1 μCi I-125 was mixed with an equal volume of 5-nm gold nanoparticles, and the mixture was left at room temperature for 16 h. A control sample contained iodine-125 labelled 15 nm gold only. The nanoparticle preparations were centrifuged at 15,000 rpm for 20 min, long enough to sediment only the 15 nm gold particles but not 5 nm size. Both pellet and supernatant fractions were counted for iodine content and OD_520_ of the supernatant. In another experiment, 0.5 ml ml of 5 nm gold was labelled with I-125 and mixed with 0.5 ml of 15 nm gold for 16 h and processed as before.

### Binding assays

Various binding assays were undertaken to demonstrate how the iodine labelling of gold nanoparticles could be used to quantify the nanomaterials in bioassays. Three types of binding assays were undertaken. Microtitre plate binding assays were done by coating microplates with ferritin and challenging with radiolabelled gold nanoparticles coated with rabbit anti-ferritin antibody or anti-ferritin and anti-rabbit combinations in an amplified assay procedure. Magnetic bead assays were done by challenging magnetic Protein A beads with a variety of radiolabelled immunogold nanoparticles. Sepharose bead binding assays were done by challenging various affinity beads with radiolabelled immunogold and comparing the percentage of radiolabel binding with the optical density (OD_520_) of gold binding.

### Post-labelling dot blots

One-centimetre square nitrocellulose dot blot assays were used to demonstrate how post-labelling may be used as a technique for quantifying gold particle binding. Mouse IgG target protein was dotted (1 to 4 μg amounts; 1 mg/ml concentration) on nitrocellulose and blocked with 2% PBS/BSA. Goat anti-mouse IgG gold (GAM gold) nanoparticles were incubated with these blots to bind to the protein. They were either pre-labelled with I-125 or not. In the case of the latter, after unbound gold was washed away, free I-125 was incubated with the blots in a post-labelling experiment. The amount of gold bound was estimated by post-labelling with I-125 and using a standard curve with increasing amounts of GAM gold directly blotted onto nitrocellulose and probing with free I-125.The nitrocellulose squares were washed and counted using the gamma counter.

## Results

### Gold nanoparticle preparations

**Fig. 1:**
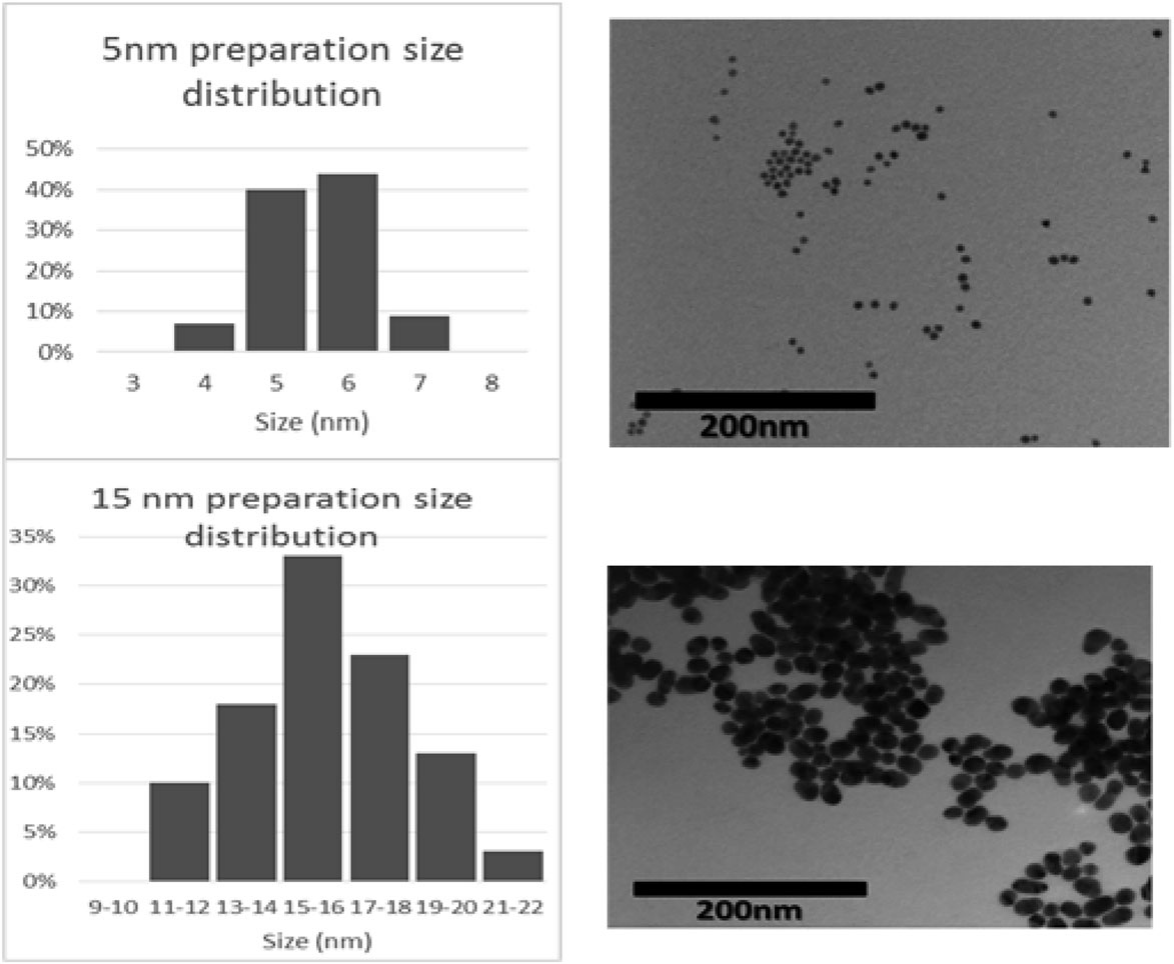
TEM images of nanoparticle preparation and size distribution

**Fig. 2:**
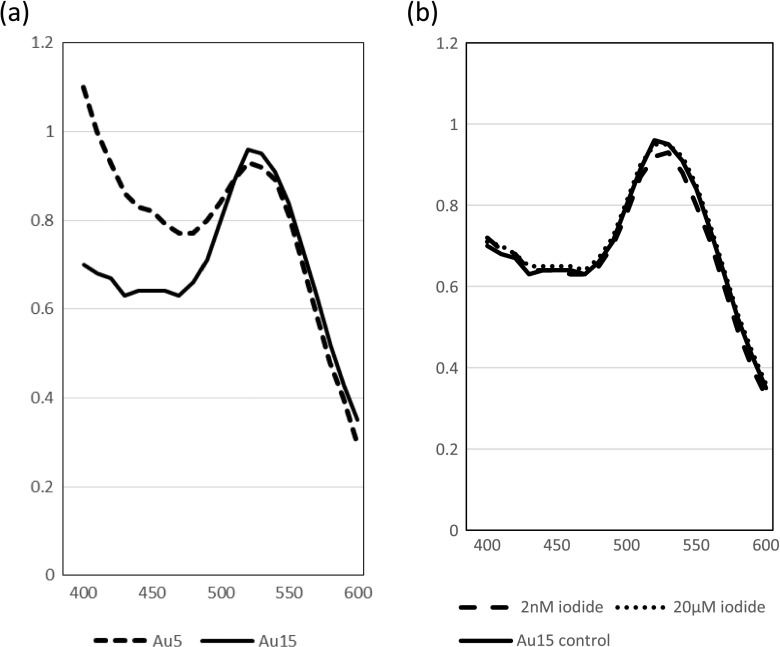
**a** Absorption spectra for gold nanoparticles. OD_max_ = 520 nm. **b** Fifteen-nanometre gold spectra with and without iodide

The preparations of nominal sizes 5 and 15 nm gold resulted in mean sizes of 6.6 and 16.4 nm, respectively, after measuring diameters of TEM images of the particles (*n* = 100) (Fig. 1). The absorption spectra indicated a plasmon resonance maximum at 520 nm with an OD = 1 (Fig. 2a). From the mean diameter sizes, the number of particles/ml were calculated as 17.4 × 10^12^ (mean size 6.6 nm) and 1.1 × 10^12^ (mean size 16.6 nm).

### Effect of iodine binding on the gold nanoparticle plasmon absorption band

Increasing the nonradioactive iodide concentration mixed with gold nanoparticles from 2 nM to 20 μM in tenfold concentration steps did not alter the plasmon absorption band at all. The profile after increasing iodide concentration four orders of magnitude was exactly the same as the control and gave a similar absorption maximum of 520 nm. Figure 2b shows the effect of 2 nM and 20 μM iodine on the plasma absorption band.

### Gold nanoparticle labelling with I-125

**Fig. 3:**
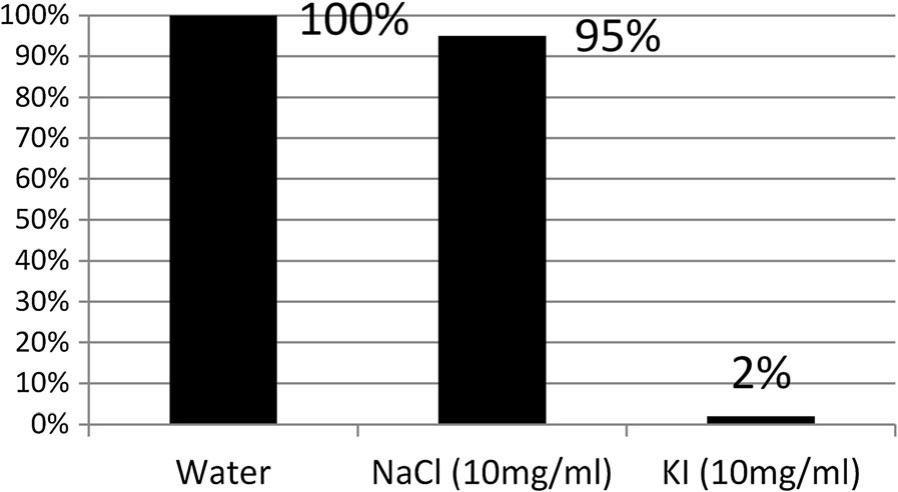
Percentage of initially bound iodine-125 to Au-15 Carbowax20M remaining after dialysis for 16 h against different solutions

Observing the mixing of iodine-25 with gold nanoparticles over different time periods indicated almost instant labelling. For consistency of results, a contact time of 30 min was allowed. Table 1 shows that labelling of gold nanoparticles is maximal (around 80% and over) regardless of whether the nanoparticles are pre-coated with molecules or not. Gold particles in water give a slightly higher yield (up to 95%) than gold in PBS. The coating molecules, whether adsorbed proteins or thiolated PEG monolayers, still allowed high efficiency of I-125 adsorption. To demonstrate that this high-affinity binding was chemisorption of iodine and not ionic binding, particles were labelled with iodine-125 and dialysed overnight against distilled water or solutions of either NaCl or KI (at 10 mg/ml). Particles retained 90% iodine-125 in the presence of water or sodium chloride but less than 2% in the presence of potassium iodide (Fig. 3).

**Table 1:** Percentage binding of 1 μCi iodine-125 to 1 ml 15 nm gold nanoparticles (OD520 = 1) coated with various molecules in water and PBS

Gold conjugate	% Iodine-125 binding in water	% Iodine-125 binding in PBS
Au-15-Uncoated	98%	Gold not stable
Au-15-BSA	96%	78%
Au-15-PEGSH	95%	82%
Au-15-Dextran	92%	78%
Au-15-Carbowax20M	90%	84%
Au-15-GAMIgG	89%	78%
Au-15-Protein A	90%	78%

Chemisorption was allowed for 30 min before centrifugal washing at 15,000 rpm for 20 min

#### Effect of ionic strength of medium

The effect of the ionic strength of the solution containing the gold nanoparticles prior to iodine labelling does not stop chemisorption, although with higher ionic strength, there is a trend towards less iodine binding (Table 2). This is probably due to competition for space of anions around the gold nanoparticle surface. The one condition that did affect the efficiency of iodine loading was the pre-dialysis of the naked gold nanoparticles. This resulted in only a 15% uptake compared with a 90% uptake for nondialysed gold. It is assumed that in an environment with minimal ions present due to dialysis, the negative charge on the iodide ions means they cannot approach the surface of the nanoparticles due to their repulsive negative charge and so chemisorption is very inefficient.

**Table 2:** Percentage of iodine-125 binding to Au-15 Protein A in the presence of variable NaCl concentrations

%NaCl	% Iodine125 binding post NaCl washes	% Iodine-125 binding in presence of variable NaCl concentrations
0	85	85
1	76	75
2	74	65
3	75	59
4	76	53
5	75	54

Addition of NaCl washes post-iodine labelling had little effect. Addition of iodine in the presence of increasing NaCl concentrations resulted in a slight reduction of the bound isotope

#### The effect of iodine-125 concentration on binding to gold

**Fig. 4:**
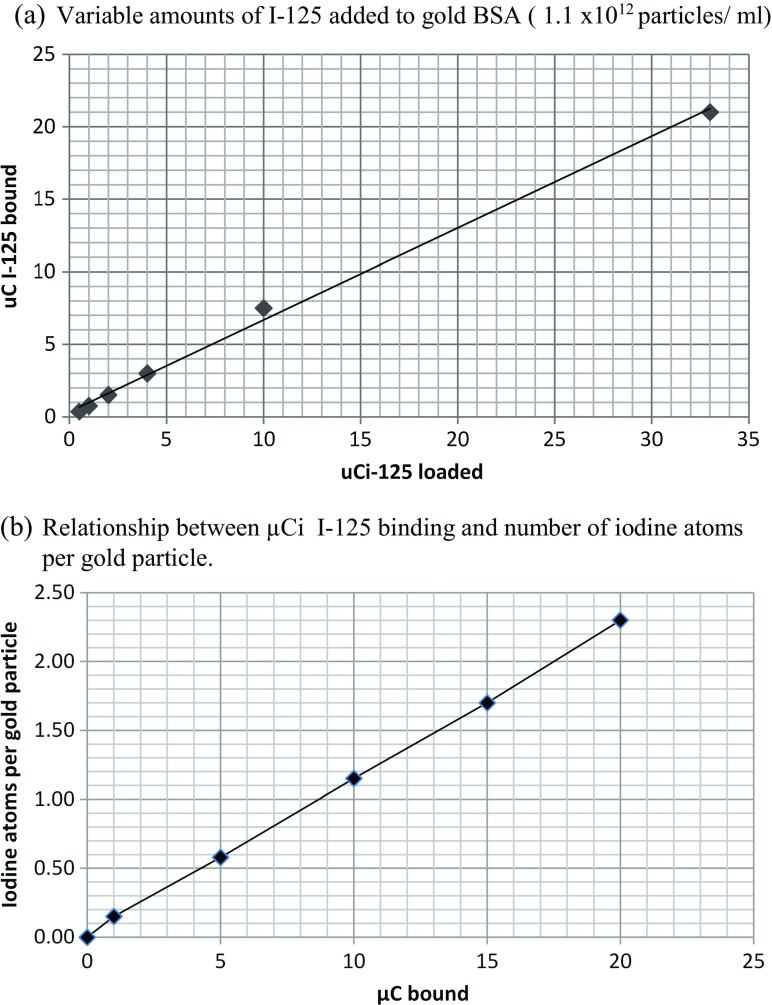
Loading of I-125 onto 15 nm gold BSA. **a** Variable amounts of I-125 added to gold BSA (1.1 × 10^12^ particles/ml). **b** Relationship between μCi I-125 binding and number of iodine atoms per gold particle

The effect of the concentration of I-125 on binding to gold nanoparticles was examined, and it was seen that binding was linear over the range tested from 1 to 30 μCi (Fig. 4a). However, on the analysis of the number of bound iodine atoms per particle, it was calculated that at these concentrations of 15-nm gold particles (1 ml, OD_520_ = 1), it needed about 9 μCi I-125 to bind to give a ratio of one atom per particle (Fig. 4b). Typically for the binding assays described later, there was probably only one iodine atom or less per particle.

### Desorption challenges to I-125 from gold nanoparticles

A variety of chemicals were used to try and desorb the radioactive iodine atoms from the gold nanoparticles by incubating 0.5 ml of radiolabelled gold-BSA with an equal volume of these solutions for 2 h before centrifugal washing (Table 3). Clearly the most effective was potassium iodide, even at very dilute concentrations. At 10 μM KI, there were signs of some desorption (<50%) and nearly total desorption at 1 mM (Table 4). High salt conditions (0.15 M sodium chloride) had minimal effect. Culture media like DMEM (a cocktail of 8 salts, 15 amino acids, 8 vitamins and glucose) and Hanks Buffer had no effect which suggests that using this labelling technique is suitable for in vitro cell culture studies. High concentrations of thiolate molecules (β mercaptoethanol) had a strong desorptive effect, a point worth noting if using additional mercaptoethanol in culture media. Excess protein in Tris/BSA buffer had no effect.

**Table 3:** Desorption of iodine-125 from gold nonoparticles by various solutions

Desorbing solution	% Iodine-125 remaining bound
Water	95
PBS	96
DMEM culture medium	92
Hanks Buffer	93
PEG-SH	95
Tris/BSA	92
KI (1 mM)	1
β-Mercaptoethanol (1 mM)	5

**Table 4:** Concentration affect of KI and β-mercaptoethanol on desorption of I-125 (value is % I-125 retained)

Concentration μM	KI	ß -Mercaptoethanol
0	92	89
1	82	82
10	76	13
100	4	7
1000	1	5

### Interparticle exchange

Pre-labelling 15-nm gold nanoparticles with I-125 and then co-incubating with nonlabelled 5 nm gold overnight resulted in no exchange to the smaller particle after separating the particles by centrifugation. When the label was on the smaller particle, there was no transfer to the larger particle (Table 5). Calculating the parking space per iodide atom on the different sizes of particle, it is clearly seen that at these ratios, there are vastly more particles than iodide atoms and the concept of an adsorbed monolayer is not appropriate.

**Table 5:** Interparticle exchange of bound iodine-125 atoms

Percent count bound						
Remaining on Au-15 after 20 h mixed with Au-5				98%		
Remaining on Au-5 after 20 h mixed with Au-15				93%		
Detail analysis: 1 μCi I-125 (50 pg) mixed with 0.5 ml gold						
	Iodine bound	Particles	Area/particle	Total surface area (nm^2^)	Area per iodine atom (nm^2^)	Particles per iodine atom
Au-15	0.98 μCi (49 pg)	0.55 × 10^12^	845 nm^2^	464 × 10^12^	2017	2.4
Au-5	0.98 μCi (46 pg)	8.7 × 10^12^	136 nm^2^	1183 × 10^12^	5377	40

I-125 was mixed with either Au-15 of Au-5 separately and allowed to adsorb. Then, unlabelled gold of the alternative size was mixed, so that each mix contained 5 and 15 nm gold, but only one was I-125 labelled. After a contact time of over 20 h, the particles were centrifuged to only sediment the 15–nm gold particles. Both fractions Au-15 (pellet) and Au-5 (supernatant) were counted for bound I-125. One picogram I-125 = 0.008 pmol; 1 pmol = 6.022 × 10^11^ iodine atoms

### Long-term stability of label

The long-term stability of iodine-labelled gold particles was examined by making 15 nm gold-Protein A and storing in a variety of buffers over a period of 100 days at 5 °C. Aliquots were taken periodically, spun down and the percentage bound calculated. Regardless of storage buffer—0.01% Carbowax in water (low ionic strength), PBS (physiological ionic strength) or 5% BSA in PBS (high protein concentration)—the iodine remained bound for up to 100 days stored at 5 °C (Table 6).

**Table 6:** Long-term stability of iodine-125 bound to gold nanoparticles in different buffers

% I-125 retained on gold particles after long-term storage			
Day	0.01% CW	PBS	5% BSA/PBS
1	92%	93%	97%
3	94%	96%	96%
7	96%	95%	94%
14	92%	91%	92%
48	89%	94%	94%
56	91%	94%	94%
74	89%	88%	93%
106	92%	94%	92%

The radiolabelled gold particles were stored at 5 °C in three different buffers for over 100 days. Samples were taken periodically, spun down and the percent of bound iodine was calculated

### Binding assays

#### Microtitre plate binding assays

**Fig. 5:**
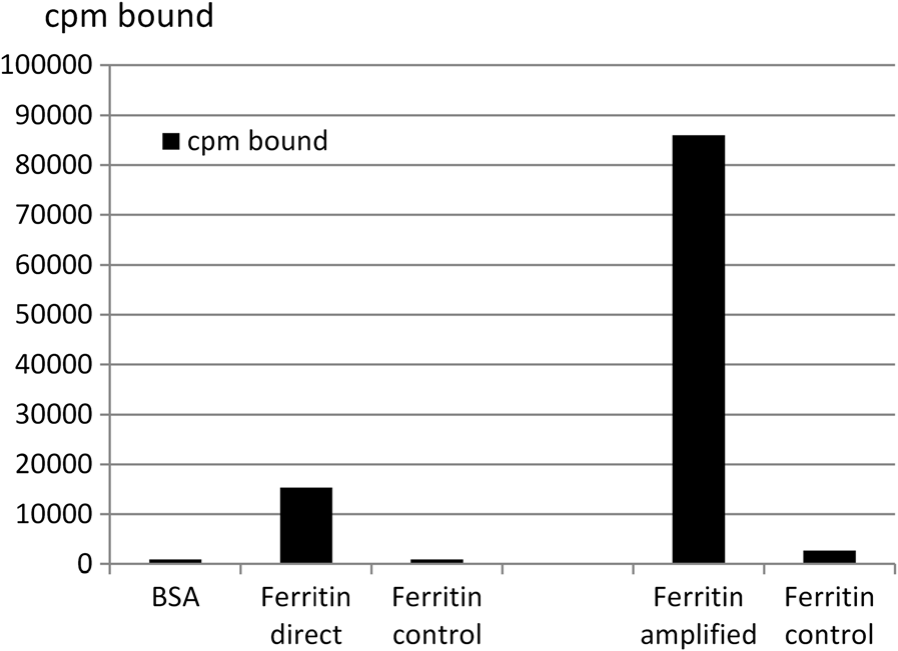
Comparison of a direct and an amplified microtitre plate binding assay of iodine-125-labelled gold nanoparticles. Wells were coated with either BSA or ferritin and probed with radiolabelled gold coated with rabbit anti-ferritin. Direct binding was demonstrated by gold anti-ferritin binding to the plate in a one-step procedure. An amplified binding assay was demonstrated by probing the plates with rabbit anti ferritin followed by goat anti-rabbit serum then by gold nanoparticles labelled with rabbit anti-ferritin. Controls were undertaken with a competitive assay format using 50 μg ferritin in solution for the first step

**Fig. 6:**
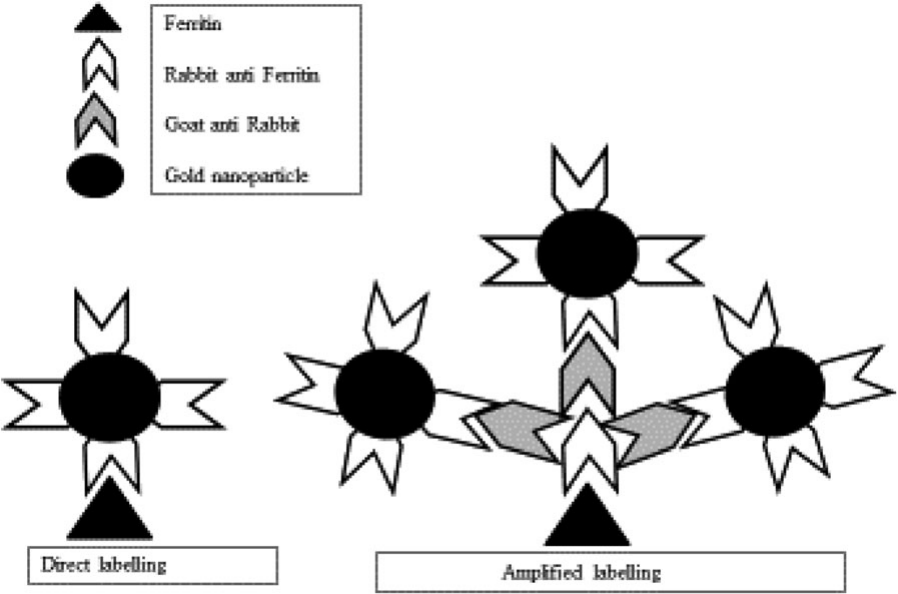
Degree of labelling depends on the protocol adopted

Binding assays in the form of a microtitre plate assay compared radio-gold binding in both a direct and amplified assay (Fig. 5). Gold particles were coated with rabbit anti-ferritin antibodies and iodine-125 (post-wash activity of 280 k cpm per assay). Direct binding was demonstrated when the plate wells, coated with ferritin, bound a total of 17 k cpm (6% of loaded) of the radiolabelled gold anti-ferritin. For the amplified binding assay, the plate bound ferritin was probed with rabbit anti-ferritin, followed by goat anti-rabbit serum and then gold particles coated with rabbit anti-ferritin, resulting in a total bound of 86 k cpm (31% of loaded particles.) The fivefold increase in binding activity is not simply due to passive versus active binding of the gold probe but also due to the multivalent nature of the polyclonal antibodies building up layers in this latter process. Figure 6 shows the schematic arrangement of this classic immunolabelling amplification technique. It demonstrates the point that for a fixed amount of labelled radio-gold, different amounts can be shown to bind quantitatively to a target.

#### Sepharose beads

When gold particles coated with Protein A and iodine-125 were mixed with Sepharose beads coupled to rabbit IgG or protein A, the binding was much more efficient than to a flat surface such as a microtitre plate. In addition, it was possible to collect the nonbound supernatant and compare the quantitative binding of radioactivity with the quantitative binding based on optical density at 520 nm. At this wavelength, relative gold particle concentrations can be compared from the same preparation and coating. Table 7 confirms that there is a close match between the radioactive and optical density data for gold particle binding to the beads. A total bound of >80% is seen in both cases compared to a nonspecific value of 3%.

**Table 7:** Correlation between radio-gold binding to Sepharose beads and OD_520_ units

Solid-phase beads	% I-125 binding	% OD_520_ binding
Sepharose rabbit IgG	92	82
Sepharose Protein A	3	2.5

Protein A gold labelled with mixed with both Sepharose-RIgG and Sepharose-PA (control). The percent binding of radiolabel was compared with the percent of OD_520_ units binding as measured by loss of these units from the supernatant

#### Magnetic bead assay

As iodine bound to gold via chemisorption, it became clear that labelling of the particles did not have to be done prior to the experiment (pre-labelling) but could possibly be done after the binding experiment (post-labelling), i.e. in situ, provided the iodine has access to the particle surface. A binding assay was set up where the aim was to couple a variety of molecules to gold particles and compare their binding efficiency to magnetic beads coupled to the target (Protein A) where pre-labelling could be compared with post-labelling. The experiment was designed to have approximately the same amount of iodine-125 per assay tube (Table 8). The results showed that post-labelling does offer possibilities as results between the two were similar. The actual counts in the post-labelling experiments were actually lower, and this is probably due to a concentration effect as the starting ratios of iodine to gold particles determine the final binding rate. It would not be possible to compare data of pre-labelled experiments with post-labelled experiments directly without a standard curve as the starting ratios of iodine and gold affect the final outcome as indicated by Fig. 4a previously.

**Table 8:** Percent count bound to magnetic beads containing Protein A

Gold reagent	% Bound (pre-labelled)	% Bound (post-labelled)
Rabbit IgG	58	46
Mouse IgG	42	38
Goat anti-mouse IgG	41	36
BSA	6	5
PEG	4	3
Con A	4	4
I-125 control	5	3

Pre-labelled were gold particles treated with I-125, washed, then added to the magnetic beads. Post-labelled were gold particles mixed with the magnetic beads first, washed and then with I-125

#### Post-incubation labelling dot blot

**Fig. 7:**
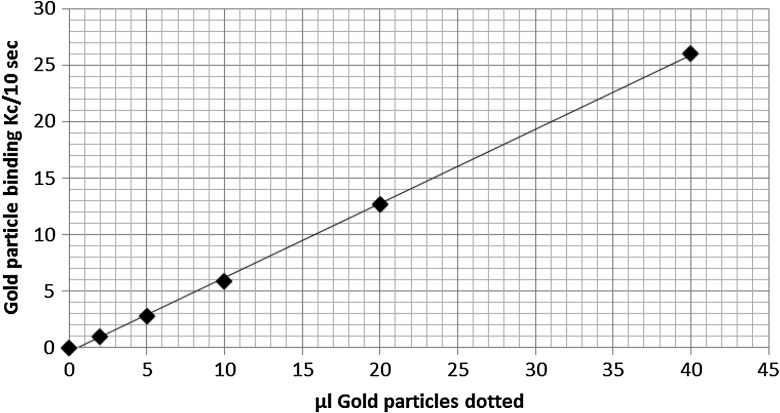
Graph showing a standard curve for post-labelling of gold particles on dot blots with I-125. Increasing volumes of GAM gold were dotted and probed with I-125 (60Kc/10s)

Post-labelling would require some form of standard curve to make sense of the bound counts. A simple assay to demonstrate this was done by incubating dot blots with variable amounts of mouse IgG and treated with a fixed amount of goat anti-mouse IgG gold either labelled with iodine-125(pre-labelled) or followed by free iodine-125 (post-labelled). In the case of the latter, a standard curve was set up by dotting increasing volumes of GAM gold directly onto nitrocellulose squares and post-labelling them with iodine-125 as well. Using this standard curve, it was possible to calculate the amount of gold binding to the mouse IgG dot blots and compare it with the amount calculated based on percent bound in the pre-labelled assay. The counts binding to the dot blots increased with the amount of IgG in both cases and in agreement with each other showing that a simple quantitation of bound gold particles using % binding in the case of pre-labelling or a standard curve in the case of post-labelling is possible. The pre-labelling assay gave a mean value of 1.4 × 10^10^ gold particles per μg mouse IgG whilst the post-labelling gave a mean value of 1.1 × 10^10^ gold particles per μg mouse IgG (Table 9 and Fig. 7).

**Table 9:** Comparison of pre- and post-labelling of gold nanoprticles on dot blots

(a) GAM gold used per assay was 0.25 ml (2.7 × 10^11^ particles) with bound I-125 (85 Kc/10 s)			
Mouse IgG (μg) dotted	Pre-labelled GAM gold-I-125 Kc/10s	% Bound	Number of gold particles binding × 10^10^
0	1	1.2%	0.3
1	5	5.9%	1.6
2	11	12.9%	3.4
3	15	17.6%	4.7
4	16	18.8%	4.9
Mean/μg	4.3	5.04%	1.4
(b) GAM gold used per assay was 0.25 ml (2.7 × 10^11^ particles), followed by I-125 (65 Kc/10s)			
Mouse IgG (μg) dotted	Post-labelled GAM gold-I-125 Kc/10s	μL equivalent of GAM gold from standard curve	Number of gold particles binding × 10^10^
0	2	3	0.4
1	7	11	1.8
2	15	24	2.6
3	22	34	3.7
4	28	44	4.7
Mean/μg	6.4	1.05	1.1

## Discussion

Gold nanoparticles have been a very useful imaging tool in electron microscopy for over 40 years. Although they can be used in a semi-quantitative way as they can be physically counted as punctate dots, this method is complicated and time consuming and not necessarily accurate. A more accurate but also time-consuming method is ICPMS. Quantitation cannot be done in situ with this method as samples have to be taken and processed elsewhere. Lab-based chemical analysis of elemental gold content is possible as in the fluorescent quenching method discussed in the introduction. However, this again requires sample digestion and further processing.

A quick and real-time method for quantitation of gold nanoparticle distribution in experimental samples is desirable. One such method that was developed 30 years ago and involved the adsorption of iodine-125 to gold nanoparticles was the first publication of a method to track and quantitate uptake of gold nanoparticles in cells in real time (Beardmore et al. 1987). Gold particles labelled with either transferrin or monoclonal anti-transferrin receptor antibody (ATR) and I-125 by chemisorption were incubated with A431 cells. After allowing for particle uptake, the cells were counted for bound radioactivity, homogenised and fractionated by density centrifugation. The fraction containing the radiolabelled gold was shown to also contain the cells’ transferrin receptor and allowed for it to be tracked and purified 200-fold in one step. The nature of the chemisorption of iodine-125 to gold was never fully explored at the time as the use of radiolabels in electron-immunocytochemistry was not deemed compatible due to the hazardous nature of the radiochemical. With the development of potential applications of gold nanoparticles in nanomedicine, it seems appropriate to fully characterise the relationship of iodine-125 chemisorbed to gold nanoparticles to help track, image and quantify the particles in experimental use.

The uptake of iodine-125 by gold nanoparticles by chemisorption is shown to be both quick and efficient in these experiments. Gold particles take up to 90% of the label almost instantly at the concentrations tested when in low ionic strength liquid. When coated gold particles were in PBS with either proteins or thiolated PEG monolayers, they took up the label at a slightly reduced level (approximately 80%). The presence of a high concentration of chloride ions had little effect on the uptake of iodide, indicating that it was indeed chemisorption. This was confirmed when it was found that as little as 100 μM KI could remove the radiolabel from gold particles but not 150 mM NaCl. Similarly, small thiolated molecules such as β-mercaptoethanol which also chemisorb to gold surfaces removed the label at 100 μM. Interestingly, loading of iodine-125 to freshly dialysed gold nanoparticles against distilled water resulted in a low efficiency take up. This may be due to changes in the ionic shell called the electrical double-layer around colloidal particles. The interpretation was that the two negatively charged entities, i.e. nanoparticle and iodide ion, were mutually repelled enough due to an expanded electrical double-layer around the particles to only allow minimal chemisorption to occur. In a medium of higher ionic strength, contact would be greater due to compression of the electrical double-layer around the particles allowing for efficient chemisorption.

Interestingly, when the ratio of bound iodine atoms per particle was calculated, it was only in the region of approximately 1:1 despite the almost total binding of the label. The initial concept of forming a complete monolayer was discounted when it was realised that the relative starting ratios of iodine to particles at the concentrations chosen would not allow this. By increasing the starting ratio of iodine to gold particles, it is possible to increase the final specific activity depending on the desired activity required. At the low ratios of iodine to gold discussed here, there is still sufficient signal to be useful as a quantitative probe. There is also the capacity to increase the ratio for higher specific activity in bio-imaging applications. It is worth noting that too high a concentration of iodine will cause gold particles to dissolve as it is a known etchant. It has been stated that KI/I solution at 0.34 mM will dissolve gold nanoparticles (Cho et al. 2009). Cheng et al. (2003) showed that KI/I at concentration of 0.2 M caused gold particles to change shape and eventually coalesce. It would seem chemisorption of iodine to gold up to an optimal loading could be desirable but beyond a certain loading could be destabilising. Work by Kim et al. (2012) showed that labelling PEG-gold particles with nonradioactive iodine at 500 μM caused a slight shift in the plasmon resonance max from 520 to 521 nm. The concentration of I-125 in our experiments are so low (2–100 nM range) that no observable change to the absorption spectra was visible.

At the concentrations used here, the bound label would appear to be very stable under physiological conditions. There does not appear to be interparticle exchange of label overnight between 5- and 15-nm gold particles. Also, the label remains bound (>90%) after 100 days when stored in three different solutions (0.1% Carbowax, PBS, and PBS containing 5%BSA). High ionic strength buffer (150 mM NaCl) does not appear to desorb the label nor does standard tissue culture medium (DMEM, Hanks Buffer). This indicates that labelled gold is suitable for quantitative work in in vitro cell uptake experiments. However, competing chemisorbing ions or molecules (free iodide or mercaptoethanol) can strip the label at >100 μM. If these labelled nanoparticles were to be used in vivo, then the possibility of desorption would have to be considered. Physiological levels of free iodide have been quoted as up to 6 μg/l or 50 nM but mainly exist as organically bound iodine at 50 μg/l (Documenta Geigy Scientific Tables 1970). At these levels of endogenous iodide, the iodine should remain bound to the gold particles. Physiological levels of free small thiolated molecules like glutathione are reported as 354 mg/l (1 mM) (Documenta Geigy Scientific Tables 1970). Research published by Shao et al. (2011) has shown that PEGylated gold nanorods labelled with I-125 survived for up to 6 days in blood circulation in rats using gamma imaging to confirm the stability of these probes in vivo.

An interesting observation relating to the stability of the bound iodine-125 was that the label was not transferred from one particle to another when particles of two different sizes were mixed. Regardless of whether the label was on a 5- or 15-nm particle, it remained there after co-incubation overnight. This could be exploited in double-labelling experiments with different sized gold particles.

Various binding assays were carried out to demonstrate how the label could be used to quantify the fraction of gold particles binding to a substrate in real time. Sepharose beads were used to demonstrate that the amount of gold particles bound as calculated using the radioactive label was equivalent to the amount of gold binding as measured by optical density at 520 nm. Magnetic beads and dot blots were used to demonstrate that post-labelling could be used to calculate amount of gold binding as long as a standard curve was included. This will allow the rapid quantitation of gold nanoparticles binding to cells in cell uptake assays providing all gold particles taken up are accessible or made to be accessible by some later process. There is lots of work currently undertaken to challenge cells with gold nanoparticles to study their toxicity. It would be more useful to relate this data to a known amount of gold taken up. Obviously, in these types of studies, there would have to be identical parallel assays where one is labelled for quantitative purposes and the other is for monitoring toxicity.

Other potential uses for iodine-125-labelled gold nanoparticles could be as a diagnostic imaging agent due to it being a gamma radiation emitter. The imaging technique known as single photon emission computed tomography (SPECT) has been proposed as a potential application. A number of papers have been recently published that highlight this approach and show how these radiolabelled nanomaterials can be used for in vivo whole animal imaging (Chrastina and Schnitzer 2010; Shao and Agarwal et al. 2011; Shao et al. 2011; Agarwal et al. 2011; Liu et al. 2015; Su et al. 2015). The work by Chrastina, using I-125-labelled silver nanoparticles, showed that by injecting a dose of 20 μCi into rats, strong visual images of localisation in the liver by SPECT could be observed even after 24 h. One of the problems of in vivo preclinical imaging using chemisorbed iodine on gold particles is that physiological iodide could assist in leaching radio-iodine from the particles if the concentration were high enough. The reported value of physiological iodide falls below the concentration required to desorb the radioactive tracer. Conversely, this property of reversible ligand exchange could be employed to strip the nanoparticles of the radiolabel once an image has been taken so that the patient is not exposed to radiation longer than is necessary.

As the chemisorption process is based on the affinity of iodine for gold, it is possible to substitute other iodine isotopes for different applications. Use of iodine-131, which is a beta emitter with a half-life of 8 days, is a more appropriate isotope for delivering a targeted therapeutic dose to a tumour for example.

Another clinical imaging technique is positron emission tomography (PET) which images positron emitting radioisotopes. This is considered to give better contrast and spatial resolution than SPECT, but the equipment is more expensive. Iodine-125 is not suitable for PET as it emits gamma radiation. However, iodine-124 emits positrons and has a half-life of 4 days which makes it ideal for clinical investigations. Imaging with targeted I-124 gold nanoparticles could be a possibility in the future.

Another technique that could be enhanced with iodine-labelled gold tracers is to quantify the uptake of gold nanoparticles into tumours in the experimental therapy known as nanoparticle enhanced radiosensitisation as demonstrated by Hainfeld et al. (2004). The presence of heavy metal nanoparticles in tumours has been shown to enhance the destructive power of conventional radiotherapy by the creation of an additional secondary radiation due to their interactions. The problem with this technique is that there is no simple way to image and quantify tumour uptake in real time. Radiolabelling gold with iodine-125 (for SPECT) or I-124 (for PET) as a tracer should allow the imaging and quantification of uptake of targeted gold to optimise the therapeutic efficiency of this potential technique.

Several recent reviews have been published giving an overview of the versatility of gold nanoparticles and their potential roles in nanomedicine (Dreaden et al. 2012; Jans and Huo 2012; Doane and Burda 2012; Azzazy et al. 2012; Jiang et al. 2012; Kumar et al. 2011). For these materials to be successful as diagnostic and therapeutic agents, it is important that they can be imaged and quantified easily. It should be possible by using the same probe of radiolabelled targeted gold nanoparticles to quantify, image and localise biological targets at three different levels of magnification. The use of the radiolabel with SPECT or PET imaging can produce images at the whole animal level. Cellular imaging is possible at the light microscope level using the silver enhancement technique and subcellular imaging at the nanoscale is possible using immunoelectron microscopy.

Just as exploiting the chemisorption of thiols and thiolated molecules onto gold surfaces enriched the use of gold nanoparticles, it is hoped that the chemisorption of iodine onto gold can be utilised in a similar way.

## Conclusions

Iodine has a high affinity for gold nanoparticles via chemisorption to the particle surface. Labelling is quick and efficient simply by mixing the two components with no complicated chemistry involved. However, low levels of free iodide will remove the isotope by ligand exchange. Pre-coated gold particles with proteins or thiolated polymers can still be labelled with iodine-125. This lends itself to developing post-labelling assays if this is advantageous. Using the concentrations of I-125 described, it is possible to label the nanoparticles with approximately only one iodine atom per particle which is well below the threshold where the nanoparticle structure becomes unstable. The labelled product is robust and stable in standard in vitro buffers and solutions with high ionic strength and concentrated proteins. The labelled particles can be used for real-time quantitation of particles in various assays. Interparticle exchange of isotope does not appear to occur. With a variety of iodine isotopes (I-124, I-125, I-131) with different properties of half-lives and radio emissions there is great potential to create targeted nanoparticles with a range of imaging and therapeutic properties in vivo.
